# OTUD7B Stabilization by METTL14-Mediated m6A Methylation Drives HIF-1**α** Expression in Esophageal Squamous Cell Carcinoma

**DOI:** 10.32604/or.2025.061301

**Published:** 2025-07-18

**Authors:** Fei Ren, Yansen Cai, Yang Song

**Affiliations:** 1 Laboratory Animal Centre, Changzhi Medical College, Changzhi, 046000, China; 2 Department of Cell Biology and Genetic, School of Basic Medical Sciences, Southwest Medical University, Luzhou, 646000, China

**Keywords:** Esophageal squamous cell carcinoma (ESCC), N6-methyladenosine (m6A), ovarian tumor deubiquitinase 7B (OTUD7B), methyltransferase-like 14 (METTL14), hypoxia-inducible factor-1α (HIF-1α), ubiquitination

## Abstract

**Objectives:**

Epigenetic changes, particularly N6-methyladenosine (m6A) modifications, play a pivotal role in cancer development. This study explored the role of ovarian tumor deubiquitinase 7B (OTUD7B) in esophageal squamous cell carcinoma (ESCC) in the context of m6A methylation and the hypoxia-inducible factor-1α (HIF-1α) pathway.

**Methods:**

The GSE179267 dataset was used to conduct differential gene expression analysis to identify key m6A-enriched genes. The Cancer Genome Atlas (TCGA), Cancer Cell Line Encyclopedia (CCLE), and Sequence-based RNA Adenosine Methylation Site Predictor (SRAMP) databases were used to evaluate the expression of OTUD7B in ESCC and its correlation with methyltransferase-like 14 (METTL14) and HIF-1α. The m6A content in total RNA extracted from ESCC cells was assessed using a dot blot assay. Gene-specific m6A-PCR was used to quantify m6A modifications in OTUD7B mRNA. The functional role of OTUD7B was explored using clonogenic and Transwell assays. The effect of OTUD7B on HIF-1α ubiquitination was detected using a co-immunoprecipitation assay.

**Results:**

OTUD7B was identified as a pivotal m6A-driven oncogene correlated with METTL14 and HIF-1α. METTL14 enhanced the mRNA stability and expression of OTUD7B through m6A methylation. OTUD7B overexpression counteracted the inhibitory effects of METTL14 knockdown on cell proliferation and invasion and stabilized HIF-1α by promoting deubiquitination.

**Conclusion:**

METTL14 plays a crucial role in the stabilization of OTUD7B through m6A methylation, thereby inhibiting the ubiquitin-proteasomal degradation of HIF-1α in ESCC. These findings highlight the potential of targeting the METTL14-OTUD7B axis as a therapeutic strategy for ESCC.

## 1 Introduction

Esophageal cancer (EC) is a prevalent malignant tumor of the digestive system that ranks seventh in incidence and sixth in mortality rates across the globe. EC affects approximately 570,000 individuals annually, resulting in 500,000 deaths, and is more prevalent in men compared to women [1]. The two predominant histological classifications of EC comprise esophageal squamous cell carcinoma (ESCC), originating from the stratified squamous epithelium lining the esophageal mucosa, and esophageal adenocarcinoma (EAC), which arises from the glandular epithelium. ESCC is predominant in China, constituting approximately 85%–90% of the cases [2,3]. The primary treatment approaches for ESCC are surgery, chemotherapy, radiotherapy, and immunotherapy [4,5]. Despite notable advancement in these treatment strategies, the rates of incidence and mortality remain high, primarily because of an incomplete understanding of the mechanisms underlying ESCC pathogenesis, metastasis, and recurrence. Fundamental knowledge of ESCC is essential.

Epigenetics studies heritable modifications in gene expression that do not result from changes in the DNA sequence [6]. These changes include chromatin remodeling, DNA methylation, noncoding RNA alterations, and RNA methylation [7]. Over a hundred types of RNA modifications have been identified, enriching the diversity and complexity of RNA functions and their genetic information [7,8]. The transcriptome undergoes diverse chemical modifications including N6-methyladenosine (m6A, the most abundant internal mRNA mark), N1-methyladenosine (m1A), N7-methylguanosine (m7G), 5-methylcytosine (m5C), 2^′^-O-methylation (Nm), and pseudouridine (Ψ), collectively forming a sophisticated regulatory layer that controls RNA metabolism through distinct mechanisms [9]. Notably, m6A accounts for approximately 80% of all mRNA modifications and has garnered considerable attention [10].

In 2011, mRNA analysis identified the fat mass and obesity-associated protein (FTO) as an m6A demethylase [11]. The discovery of m6A’s reversible nature ignited extensive research into its regulatory mechanisms, revealing a sophisticated system where the methyltransferase complex (METTL3-METTL14-WTAP) installs m6A modifications, while demethylases (FTO/ALKBH5) remove them, creating dynamic methylation cycles. These modifications are subsequently interpreted by reader proteins—the YTHDF1/2/3 family mediates mRNA stability and translation, while IGF2BP1/2/3 influences transcript localization and expression, collectively forming a comprehensive post-transcriptional regulatory network that rapidly responds to cellular signals and controls diverse biological processes [10]. Yang et al. [12] developed an m6A scoring system and discovered a close association between the m6A score and immune cell infiltration within the tumor immune microenvironment of ESCC; higher m6A scores were associated with poorer prognoses. Furthermore, m6A modification has been correlated with ESCC immune cell infiltration and tumor mutational burden [13], further underscoring the crucial role of m6A modification in ESCC progression.

In the present study, bioinformatics identified OTUD7B as a key gene driven by m6A methylation in ESCC. Acting as a deubiquitinating enzyme, OTUD7B modulates cellular processes such as the cell cycle, oncogenesis, neural progenitor cell differentiation, and inflammatory responses by targeting the deubiquitination of specific substrates [14]. OTUD7B is frequently amplified in various malignant tumors, including those affecting the stomach, liver, breast, pancreas, and prostate, where it promotes tumor initiation and progression. Wang et al. [15] associated higher OTUD7B expression with poorer outcomes in patients with breast cancer, linking enhanced OTUD7B expression to increased malignancy and metastatic potential. Kim et al. [16] demonstrated that OTUD7B enhances prostate cancer cell proliferation and autophagy, and inhibits apoptosis via the protein kinase B/mammalian target of rapamycin (AKT/mTOR) signaling pathway. Nevertheless, the expression profile and functional role of OTUD7B in ESCC remain unknown. Briefly, OTUD7B was selected for in-depth study based on two main factors. First, bioinformatic analysis of the GSE179267 dataset identified OTUD7B as a gene with significant m6A enrichment in ESCC tissues. This finding suggested its potential involvement in m6A-mediated regulatory pathways relevant to ESCC. Second, a review of the existing literature indicated that while OTUD7B has been studied in several types of cancers, its role in esophageal cancer remains unclear. The limited understanding of OTUD7B’s biological role in ESCC motivated our comprehensive investigation to elucidate its molecular mechanisms, with the ultimate goal of identifying novel pathogenic pathways and developing targeted intervention strategies for this aggressive malignancy. Therefore, investigating the biological functions of OTUD7B in the onset and progression of ESCC is necessary, to ascertain whether its activity is driven by m6A methylation, and to uncover the potential mechanisms.

This study investigated the m6A methylation patterns of OTUD7B in ESCC cells. RNA methyltransferases that modulate m6A enrichment were assessed and downstream target genes that may be regulated by ubiquitination were explored.

## 2 Materials and Methods

### 2.1 Bioinformatic Analysis

The Gene Expression Omnibus (GEO) repository served as the primary source for microarray data extraction. Researchers utilized the GSE179267 dataset (accessible via https://www.ncbi.nlm.nih.gov/gds/(accessed on 25 April 2025)), which was generated using the GPL23227 platform (BGISEQ-500 human genome sequencing). This particular dataset included two m6A-enriched esophageal carcinoma tissue specimens along with one control input sample. For differential gene expression profiling, we implemented the limma software suite in R (v3.5.1), applying Bayesian *t*-test statistics with stringent thresholds (absolute log2 fold change ≥1 and false discovery rate-adjusted *p*-value < 0.05). Data visualization was accomplished through multiple R packages: ggplot2 for generating volcano plots depicting m6A-modified genes, and pheatmap for constructing clustered heatmaps that displayed both upregulated and downregulated transcripts, with sample dendrograms positioned above and gene clusters aligned vertically.

To evaluate OTUD7B expression patterns, we integrated data from two major cancer databases: The Cancer Genome Atlas (TCGA; https://portal.gdc.cancer.gov/) provided tissue-specific expression profiles comparing esophageal squamous cell carcinoma vs. normal adjacent tissue, while the Cancer Cell Line Encyclopedia (CCLE; https://sites.broadinstitute.org/ccle/ (accessed on 25 April 2025)) enabled comprehensive analysis across multiple EC cell lines. CCLE results were graphically represented using a color-coded bar plot, where cell line identifiers were arranged along the vertical axis and expression levels were quantified through both bar height and chromatic intensity, using median expression as the threshold value.

For m6A site prediction in OTUD7B transcripts, we employed the SRAMP web server (http://www.cuilab.cn/sramp (accessed on 25 April 2025)). Functional annotation of the top 20 differentially m6A-modified genes was conducted through KEGG pathway analysis using R’s ClusterProfiler toolkit. Furthermore, we examined potential regulatory relationships by assessing expression correlations among OTUD7B, the RNA methyltransferase METTL14, and hypoxia-responsive factor HIF-1α in EC cells, utilizing the GEPIA platform (http://gepia.cancer-pku.cn/) for interactive expression profiling.

### 2.2 Cell Culture

The human ESCC cell lines TE-8 and TE-1, along with the non-malignant esophageal epithelial cell line HET-1A, were procured from the American Type Culture Collection (ATCC, Manassas, VA, USA). These cell lines were cultured in Dulbecco’s Modified Eagle’s Medium (DMEM, Gibco, Thermo Fisher Scientific, Waltham, MA, USA; Cat. No. 11966025) enriched with 10% fetal bovine serum (FBS, Gibco; Cat. No. 10438026) and 1% penicillin-streptomycin antibiotic mixture (Corning, NY, USA; Cat. No. 30-002-CI). Cell incubation was carried out at 37°C in a 5% CO_2_ humidified atmosphere to maintain optimal growth conditions. For experimental procedures, cells were harvested during their logarithmic growth phase to ensure consistent viability and proliferation rates. Cells grown on solid media were sub-cultured when growth was approximately 75% confluent. Cell authentication was validated using short tandem repeat profiling. To ensure that the cell lines were mycoplasma free, they were routinely tested using a PCR Mycoplasma Detection Kit (Catalog number J66117.AMJ; Thermo Fisher Scientific).

### 2.3 m6A Dot Blot Assay

RNA or poly(A)^+^ mRNA was isolated using an established protocol. The mRNA samples were resuspended in RNA incubation buffer (Catalog number 93289; Sigma-Aldrich, St Louis, MO, USA) at a volume three-times that of the original, and denatured by heating at 65°C for 5 min. The samples were then divided into aliquots containing 400, 200, and 100 ng of mRNA, and transferred to an Amersham Hybond-N^+^ membrane (Catalog number RPN303B; GE Healthcare, Chicago, IL, USA) using a Bio-Dot microfiltration apparatus (Catalog number 170–6545; Bio-Rad Laboratories, Hercules, CA, USA), along with a solution of chilled 20× SSC buffer (Catalog number J60839.K3; Thermo Fisher Scientific). The membrane was subjected to ultraviolet crosslinking for 5 min and was rinsed with 0.1 M PBS containing Tween-20. Following incubation with a 5% milk solution to block nonspecific binding, the membrane was probed overnight using an m6A antibody (1:1000 dilution; Millipore, Billerica, MA, USA) at 4°C. Subsequently, the dot blots were incubated with horseradish peroxidase (HRP)-conjugated anti-mouse IgG antibody (1:1000 dilution, Catalog number A0216; Beyotime, Beijing, China) for 60 min at room temperature prior to visualization using the ChemiDoc XRS + system (Bio-Rad).

### 2.4 Gene-Specific m6A-PCR

Cellular RNA was purified from all experimental groups using standard extraction methods. Messenger RNA was subsequently isolated from total RNA samples employing a poly(A) tail-based purification system (Thermo Fisher Scientific, #61006). To ensure RNA purity, samples were treated with DNase I (Thermo Fisher Scientific, EN0521) for complete genomic DNA removal. For m6A-modified RNA detection, we performed immunoprecipitation assays using the Magna MeRIP m6A kit (Thermo Fisher Scientific, 20164), following the manufacturer’s established protocol. This involved incubating total RNA with anti-m6A antibodies to specifically capture methylated RNA fragments. Following immunoprecipitation, we conducted reverse transcription polymerase chain reaction (RT-PCR) analysis targeting OTUD7B transcripts. Primer sequences were specifically designed to amplify regions of interest within the OTUD7B mRNA sequence. All experimental measurements were standardized against corresponding input RNA controls to ensure data reliability.

### 2.5 Overexpression and Knockdown Experiments

The full-length OTUD7B cDNA was directionally cloned into the pcDNA3.1 mammalian expression vector (GenePharma, Shanghai, China). For gene silencing experiments, we designed two distinct shRNA sequences targeting different regions of both OTUD7B and METTL14 transcripts:

OTUD7B-targeting sequences:

shOTUD7B-1: 5^′^-CGGGTTCATTGCGTCATTAAT-3^′^

shOTUD7B-2: 5^′^-ATCCTGTAAATCCTGTAAATT-3^′^

METTL14-targeting sequences:

shMETTL14-1: 5^′^-ACTCGGAGAGACGGCATTTAA-3^′^

shMETTL14-2: 5^′^-ATTTGTGCAGTGTTCTTAATT-3^′^

Negative control:

shNC: 5^′^-GTCAATGGTCGTGTCGTGC-3^′^

Prior to transfection, cells were maintained in complete growth medium until reaching 70%–80% confluence. The transfection mixture was prepared using antibiotic-containing, serum-free DMEM (serum elimination prevents interference with liposome formation, while antibiotics maintain sterility without affecting eukaryotic cell viability). Plasmid DNA or shRNA constructs were introduced into cells using Lipofectamine^®^ 3000 transfection reagent (Thermo Fisher Scientific, Cat# L3000008) according to the manufacturer’s optimized protocol. Following transfection, all cell cultures were maintained under standard conditions (37°C, 5% CO_2_ humidified atmosphere) for 24–48 h before subsequent experimental analyses. Cells were then collected for downstream applications including protein extraction or functional assays.

### 2.6 Western Blotting

Cellular proteins were isolated using RIPA lysis buffer (Beyotime, P0013B) containing protease and phosphatase inhibitors (Thermo Fisher Scientific, 78440) to maintain protein integrity. Protein concentrations were determined using the BCA assay (Thermo Fisher Scientific, 23227) according to the manufacturer’s protocol. For electrophoresis, equal amounts of protein lysates were resolved on 4%–12% SDS-polyacrylamide gels and subsequently transferred onto PVDF membranes (Millipore, HVLP02500, 0.45 μm pore size). To minimize nonspecific binding, membranes were blocked with 5% non-fat milk in TBST (Thermo Fisher Scientific, J77500.K2) for 1 h at room temperature. Primary antibody incubation was performed overnight at 4°C, followed by washing and incubation with HRP-conjugated secondary antibodies for 1 h at room temperature. Protein signals were detected using an ECL substrate (Servicebio, G2014-500ML) and captured using a ChemiDoc™ MP imaging system (Bio-Rad). β-actin was used as a loading control to ensure equal protein loading across samples. Antibody details are summarized in Table 1.

**Table 1:** Antibodies used for western blotting

Antibody	Manufacturer	Cat. No.	Dilution
Anti-METTL14	Abcam (Cambridge, MA, USA)	ab309096	1:1000
Anti-OTUD7B	ProSci (Poway, CA, USA)	57-615	1:1000
Anti-β-actin	Beyotime	AF5003	1:2000
Anti-YTHDF1	Beyotime	AF8382	1:1000
Anti-HIF-1α	Beyotime	AG2135	1:1000
HRP-labeled Goat Anti-Rabbit IgG	Beyotime	A0208	1:1000

### 2.7 RNA Extraction and RT-qPCR Analysis

Total RNA was purified from samples using the RNA-Quick Purification Kit (ESScience, RN001) following the manufacturer’s guidelines. First-strand cDNA synthesis was carried out with the HiScript II Q RT SuperMix (Vazyme, R223-01), a reverse transcription system optimized for quantitative PCR applications. For gene expression analysis, we performed quantitative real-time PCR (qPCR) using SYBR Green master mix (Vazyme, Q221-01) on a QX100 Droplet Digital PCR system (Bio-Rad). The thermal cycling conditions consisted of an initial denaturation step at 95°C for 30 s, followed by 40 cycles of 95°C for 10 s and 60°C for 30 s. Gene expression levels were calculated using the comparative Ct (2^−ΔΔCt^) method, with GAPDH serving as the internal reference gene for normalization. This approach allowed for accurate relative quantification of target gene expression across different experimental conditions. All primer sequences used in this study are detailed in Table 2.

**Table 2:** Primers used for PCR

Gene	Sequence	
METTL14 (*Homo sapiens*)	Forward	CTGAAAGTGCCGACAGCATTGG
	Reverse	CTCTCCTTCATCCAGATACTTACG
β-actin (*Homo sapiens*)	Forward	CACCATTGGCAATGAGCGGTTC
	Reverse	AGGTCTTTGCGGATGTCCACGT
OTUD7B (*Homo sapiens*)	Forward	TCTCAGAGGCTGCTTCCTTTGG
	Reverse	CGCCTTTTCAACGCTTCCTTCTC

### 2.8 OTUD7B mRNA Degradation Rate

To assess the transcriptional stability of OTUD7B mRNA, we employed actinomycin D (Act D, Sigma-Aldrich, A9415) at a working concentration of 5 μg/mL in cultured cells. This transcriptional inhibitor was added directly to the culture medium to block *de novo* RNA synthesis. Following treatment periods ranging from 0 to 6 h, total RNA was isolated from cell lysates using standard extraction methods. The extracted RNA was subsequently subjected to RT-qPCR analysis following our laboratory’s optimized protocol. This approach allowed us to quantitatively track the decay kinetics of OTUD7B transcripts over time. All measurements were performed in triplicate to ensure reproducibility, with data normalized to internal control transcripts showing stable expression throughout the inhibition period.

### 2.9 Clone Formation Assay

Logarithmically growing cells from each group were seeded into 12-well plates at a density of 1 × 10^3^ cells/well, with triplicate wells for each group. Colony formation was monitored as the emergence of visible cell colonies approximately two weeks after seeding. Subsequently, the cells were fixed in 4% paraformaldehyde for 30 min, and then stained with 0.1% crystal violet (Catalog number E607309; Sangon Biotech, Shanghai, China) for 15 min. Excess stain and nonspecific materials were carefully washed away with PBS until the colonies were clearly visible and dry. The colonies were photographed and the number of colonies was counted to determine cloning efficiency.

### 2.10 Transwell Assay

Cell invasion was evaluated using Matrigel-coated Transwell chambers (8-μm pores, Corning) in a standardized *in vitro* assay. Briefly, 5 × 10^4^ ESCC cells suspended in serum-free medium were seeded into the upper chamber, while the lower chamber contained medium with 10% FBS as a chemoattractant. After 48 h of incubation at 37°C, non-invading cells were removed from the upper membrane surface. The invaded cells on the lower membrane surface were fixed with 4% paraformaldehyde and stained with 0.1% crystal violet solution. Quantitative analysis was performed by counting stained cells in five randomly selected microscopic fields (200× magnification) per membrane using ImageJ software.

### 2.11 Co-Immunoprecipitation (Co-IP) Assay

For protein interaction studies, TE-8 cell lysates were prepared using RIPA lysis buffer (Beyotime, P0013B) containing protease inhibitors. The lysates were incubated overnight at 4°C with specific primary antibodies targeting either OTUD7B or HIF-1α. Protein A/G agarose beads (Thermo Fisher Scientific, 20421) were then added to the antibody-protein complexes and incubated for 4 h at 4°C with gentle rotation. Following incubation, the bead-bound immunocomplexes were pelleted by brief centrifugation at 4°C and washed three times with cold PBS to remove non-specific binding. The captured proteins were eluted by boiling in 2× SDS loading buffer for 5 min at 95°C. The eluted proteins were subsequently separated by SDS-PAGE and analyzed by western blotting to detect potential interactions between OTUD7B and HIF-1α.

### 2.12 Ubiquitination Assay

To investigate protein ubiquitination patterns, TE-8 cells were simultaneously transfected with two expression constructs: one encoding HA-tagged ubiquitin and another containing the full-length OTUD7B gene. After allowing 36 h for protein expression, cells were treated with the proteasome inhibitor MG132 (Sigma-Aldrich, M7449) at a working concentration of 10 μM for 6 h to stabilize ubiquitinated proteins. Following treatment, cells were harvested and lysed under denaturing conditions. The resulting lysates were subjected to affinity purification using HA-specific magnetic beads (Thermo Fisher Scientific, 88836) to selectively enrich for ubiquitin-modified proteins. After multiple stringent washes with TBST buffer to eliminate nonspecific interactions, the captured protein complexes were released from the beads by heating in SDS sample buffer at 95°C for 5 min. The eluted proteins were then resolved by SDS-PAGE and transferred to PVDF membranes for immunoblot analysis. This approach enabled the detection and characterization of ubiquitin-modified proteins and their interaction partners through subsequent probing with specific antibodies.

### 2.13 Statistical Analyses

Quantitative data are presented as mean values ± SEM from at least three independent experiments. All statistical analyses were conducted using GraphPad Prism software (v8.0, GraphPad Inc, San Diego, CA, USA). Intergroup differences were evaluated using appropriate statistical tests: two-group comparisons employed unpaired Student’s *t*-tests with Welch’s correction, while multiple group comparisons utilized one-way ANOVA followed by Tukey’s post-hoc test. A probability value of *p* < 0.05 was considered statistically significant for all analyses.

## 3 Results

### 3.1 Bioinformatics-Based Identification of OTUD7B as a Crucial m6A Methylation-Driven Oncogene in ESCC Tissues Correlated with METTL14 and HIF-1**α** Expression

**Figure 1:**
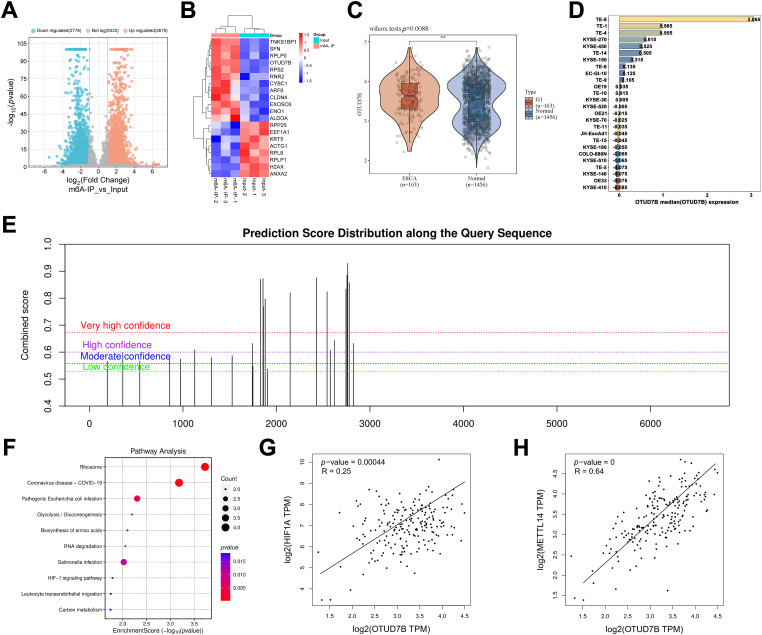
Bioinformatic analysis identifies OTUD7B as a Crucial m6A methylation-driven oncogene in ESCC tissues whose expression is correlated with that of METTL14 and HIF-1α. **(A)**: Analysis of key genes with significant m6A enrichment in esophageal squamous cell carcinoma (ESCC) cells, based on the GSE179267 dataset. **(B)**: Heatmap of the top 20 differentially m6A-enriched genes. **(C)**: Analysis of OTUD7B expression in ESCC and paracancerous tissues based on TCGA. **(D)**: Analysis of the expression distribution of the *OTUD7B* in different ESCC cell lines based on CCLE. The height and color of the bars represent the magnitude of gene expression, with the median value as the boundary point. **(E)**: Search for OTUD7B mRNA sequences in SRAMP (m6A methylation sequencing database) to analyze m6A binding sites. **(F)**: Kyoto Encyclopedia of Genes and Genomes (KEGG) enrichment analysis of the top 20 differentially m6A-enriched genes using the ClusterProfiler package in the R environment. **(G)**: Analysis of the correlation between OTUD7B and HIF-1α expression based on GEPIA. **(H)**: Analysis of the correlation between OTUD7B and RNA methyltransferase METTL14 expression in esophageal cancer (EC) based on GEPIA; **p < 0.01

The first experiment involved utilization of the GSE179267 dataset for assessment of key genes with significant m6A enrichment in ESCC cells. Fig. 1A shows a volcano plot of the 5455 identified differentially m6A-enriched genes. Among these, 2679 were significantly m6A-enriched and 2776 were not m6A-enriched. A heatmap was constructed for the top 20 differentially m6A-enriched genes, comprising TNKS1BP1, SFN, RPLP0, OTUD7B, RPS2, RNR2, CYBC1, ARF6, CLDN4, EXOSC6, ENO1, ALDOA, RPP25, EEF1A1, KRT5, ACTG1, RPL8, RPLP1, H2AX, and ANXA2, with particular interest in OTUD7B (Fig. 1B). Further TCGA analysis for assessing OTUD7B expression in ESCC tissues compared to that in paracancerous tissues revealed a notable upregulation in ECSS tissues (Fig. 1C). Examination of OTUD7B expression across diverse EC cell lines in CCLE revealed its upregulation in multiple EC cell lines, with the highest expression observed in ESCC cell lines TE-8 and TE-1 (Fig. 1D). Given the significant enrichment of m6A in OTUD7B mRNA in the GSE179267 dataset, we searched for OTUD7B mRNA sequences in the SRAMP database, an m6A methylation sequencing repository. Several m6A binding sites were identified (Fig. 1E). Fig. S1 details the m6A binding sites on OTUD7B mRNA, specifically site1829, site1856, site2146, and site2762. Subsequently, KEGG enrichment analysis was performed on the top 20 differentially m6A-enriched genes using the ClusterProfiler package in R, leading to the identification of the HIF-1α signaling pathway (Fig. 1F). This finding suggests a potential link between the HIF-1α signaling pathway and m6A enrichment of OTUD7B mRNA. The significant positive correlation between the expression of OTUD7B and HIF-1α was assessed in EC cells using GEPIA (Fig. 1G). Subsequent analysis of the correlation between the expression of OTUD7B and m6A methylation-related enzymes revealed a significant positive correlation between the expression of OTUD7B and the RNA methyltransferase METTL14 in EC tissues (Fig. 1H). The collective findings support the role of OTUD7B as a key gene whose expression is driven by m6A methylation in ESCC and exhibits a positive correlation with the expression of both METTL14 and HIF-1α.

### 3.2 METTL14 Enhances the Stability of OTUD7B mRNA and Expression of OTUD7B Protein by Regulating Its m6A Methylation

**Figure 2:**
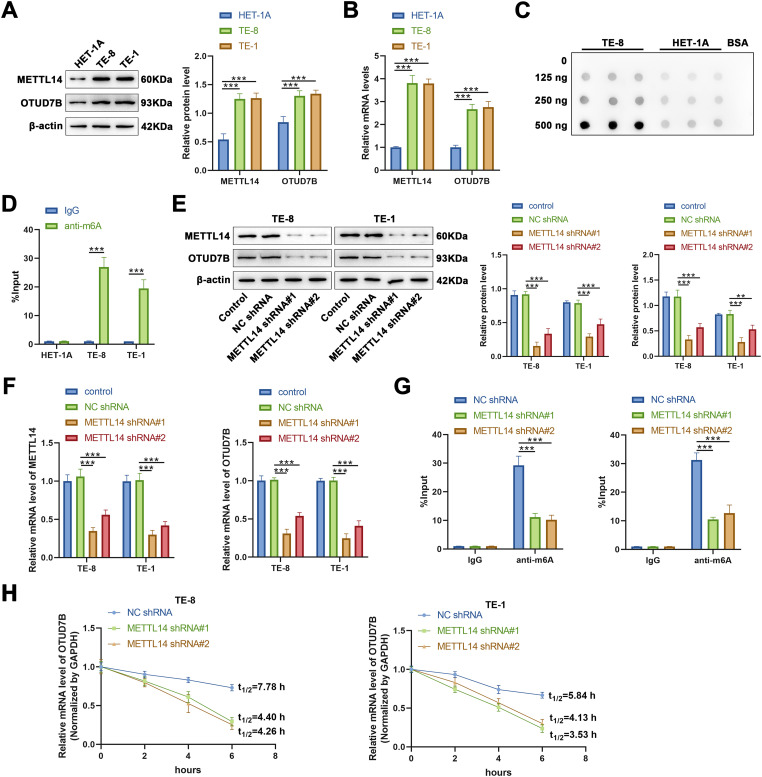
METTL14 enhances the mRNA stability and protein expression of OTUD7B by regulating its m6A methylation in ESCC cells. **(A,B)**: Western blotting (**A**) and qRT-PCR (**B**) to assess the expression of METTL14 and OTUD7B in TE-8 and TE-1 ESCC and HET-1A human esophageal epithelial cell lines. **(C)**: Dot blot analysis of m6A modification in total RNA extracted from TE-8 and HET-1A cells. RNA samples were serially diluted by a factor of two, and equal amounts of 125, 250, and 500 ng were loaded. Bovine serum albumin was used as a negative control. **(D)**: Gene-specific m6A-PCR evaluation of the m6A modification levels in specific regions of the OTUD7B transcript in TE-8, TE-1, and HET-1A cells. **(E,F)**: Cells were transfected with METTL14 shRNA#1 and METTL14 shRNA#2, followed by the assessment of METTL14 and OTUD7B expression using western blotting (**E**) and qRT-PCR (**F**). **(G)**: Post-METTL14 knockdown in TE-8 and TE-1 cells. Gene-specific m6A-PCR was employed to determine the m6A modification levels in specific regions of the OTUD7B transcript. **(H)**: Determination of degradation rate of OTUD7B mRNA at various time points using RT-qPCR after inhibiting METTL14 in TE-8 and TE-1 cells, followed by treatment with actinomycin D (Act D, 5 μg/mL). Data are expressed as mean ± SEM, *n* = 3; **p < 0.01, ***p < 0.001

To validate the aforementioned findings regarding OTUD7B obtained via database analyses, qRT-PCR was applied to first evaluate the expression of OTUD7B and METTL14 in TE-8 and TE-1 ESCC cells and HET-1A human esophageal epithelial cells. Compared to those in HET-1A cells, the mRNA levels of OTUD7B and METTL14 were markedly upregulated in TE-8 and TE-1 cells (Fig. 2A). Subsequently, the global levels of m6A in the total RNA from TE-8 and HET-1A cells was assessed by dot blotting. A marked increase in m6A levels was evident in TE-8 cells, indicating that a marked increase in m6A modification in EC may be associated with OTUD7B m6A enrichment (Fig. 2B).

To confirm the m6A enrichment in OTUD7B mRNA, gene-specific m6A-PCR was performed to detect the m6A modification levels at specific regions of the OTUD7B transcript in TE-8 and TE-1 ESCC cells and HET-1A cells. Significant m6A enrichment in the OTUD7B transcript was evident in TE-8 and TE-1 cells, but not in HET-1A cells (Fig. 2C,D).

Subsequently, to analyze whether METTL14 regulates the m6A modification on OTUD7B mRNA, two shRNAs targeting METTL14 (METTL14 shRNA#1 and METTL14 shRNA#2) were transfected into TE-8 and TE-1 cells, and the expression of METTL14 and OTUD7B was measured by qRT-PCR and western blotting. METTL14 shRNA#1 and METTL14 shRNA#2 significantly reduced METTL14 expression at both the protein and mRNA levels, indicating efficient knockdown. Moreover, the expression of OTUD7B at protein and mRNA levels was markedly decreased upon METTL14 knockdown, suggesting that METTL14 positively regulates OTUD7B expression at both the mRNA and protein levels (Fig. 2E,F).

Gene-specific m6A-PCR was used to examine m6A modification levels at specific regions of the OTUD7B transcript upon METTL14 knockdown in TE-8 and TE-1 cells. A significant reduction in m6A modification levels on OTUD7B was evident (Fig. 2G). Additionally, after treating TE-8 and TE-1 cells with Act D (5 μg/mL) following METTL14 inhibition, the degradation rate of OTUD7B mRNA at various time points was analyzed using RT-qPCR. METTL14 knockdown significantly reduced the stability of OTUD7B mRNA and shortened its half-life (Fig. 2H). To further investigate which m6A modification site on OTUD7B mRNA was involved in the regulation of OTUD7B expression by METTL14, four overexpression vectors were constructed in which the methylated adenine residue (A1829, 1856, 2146, and 2762) was replaced by guanine (A–G); 1829mut, 1856mut, 2146mut, and 2762mut, respectively. Compared with that in the control (wild type [WT] cell line), m6A enrichment on the OTUD7B transcript was significantly decreased in the 1856mut and 2762mut cell lines (Fig. S2A). The expression of OTUD7B increased and decreased in 1856mut and 2762mut cell lines, respectively (Fig. S2B,C). RNA decay assays revealed that the mutation at site 2762 resulted in increased degradation of OTUD7B mRNA (Fig. S2D). This result suggests that the stability of OTUD7B mRNA due to m6A modification is predominantly controlled by the modification at site 2762. YTH N6-methyladenosine RNA binding protein 1 (YTHDF1) regulates the stabilization and translation of specific m6A-containing mRNAs [17]. Further analysis revealed that the enrichment of OTUD7B in YTHDF2-IP decreased after the mutation at site 2762 (Fig. S2E). These results suggest that the m6A site at 2762 in OTUD7B may interact with YTHDF1 to regulate OTUD7B mRNA stability and translation efficiency.

Based on these findings, we inferred that METTL14 may promote the mRNA stability and protein expression of OTUD7B by mediating its m6A methylation. However, whether METTL14-mediated m6A methylation of OTUD7B mRNA is involved in regulating EC cell behavior remains to be determined.

### 3.3 Overexpression of OTUD7B Counteracts the Suppressive Effects of METTL14 Knockdown on ESCC Cell Proliferation and Invasion

**Figure 3:**
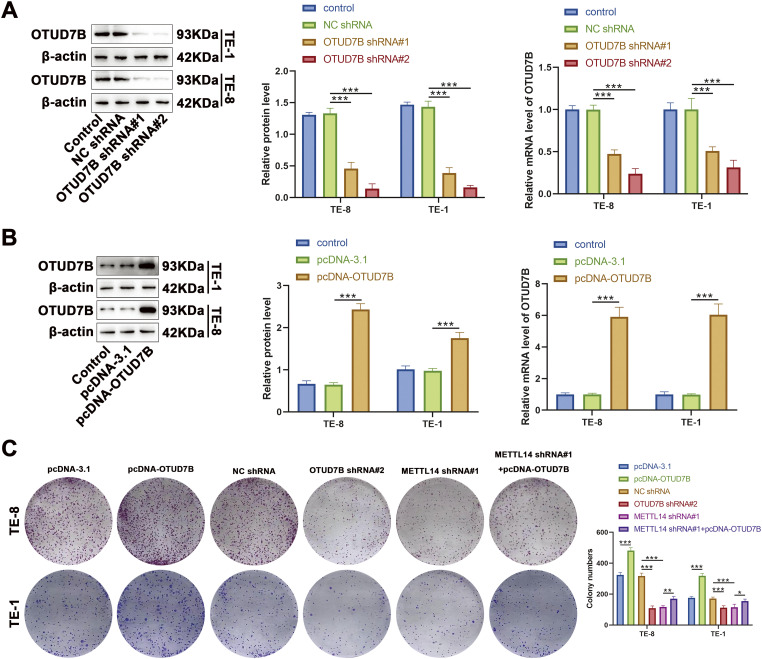

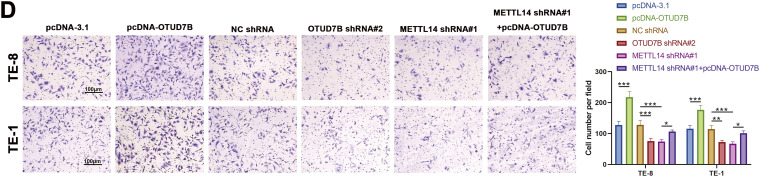
Overexpression of OTUD7B counteracts the suppressive effects of METTL14 knockdown on ESCC cell proliferation and invasion. **(A)**: OTUD7B expression assessed by qRT-PCR and western blotting in TE-8 and TE-1 cells transfected with OTUD7B shRNA#1 and OTUD7B shRNA#2. **(B)**: OTUD7B expression determined by qRT-PCR and western blotting in TE-8 and TE-1 cells transfected with pcDNA-OTUD7B. **(C)**: Clonogenic assay of the proliferative capacity of cells across the different groups. **(D)**: Transwell assay assessment of the invasive capabilities of cells in each group. Data are expressed as mean ± SEM, *n* = 3; *p < 0.05, **p < 0.01, ***p < 0.001

The influence of METTL14-mediated m6A methylation of OTUD7B mRNA on behavioral alterations was assessed in ESCC cells. Cells were first transfected with OTUD7B shRNA#1 and OTUD7B shRNA#2. The expression of OTUD7B was subsequently evaluated by qRT-PCR and western blotting. Results revealed that OTUD7B shRNA#2 exhibited better knockdown efficiency than OTUD7B shRNA#1 (Fig. 3A). Thereafter, cells were transfected with pcDNA-OTUD7B to induce OTUD7B overexpression. qRT-PCR and western blotting revealed more than 6-fold increase in OTUD7B expression post-transfection, signifying effective overexpression (Fig. 3B).

In cellular behavior assays, clonogenic and Transwell experiments revealed that OTUD7B overexpression resulted in increased colony counts and enhanced invasiveness of TE-8 and TE-1 cells. Conversely, OTUD7B knockdown resulted in fewer colonies and decreased invasive capacity. METTL14 knockdown also resulted in fewer cell colonies and invasiveness. Notably, the inhibitory effects of METTL14 knockdown on cell proliferation and invasion were significantly reversed by OTUD7B overexpression (Fig. 3C,D). These observations suggest that OTUD7B overexpression counteracts the inhibitory effects of METTL14 knockdown on ESCC cell proliferation and invasiveness.

### 3.4 OTUD7B Stabilizes HIF-1**α** Expression by Modifying Deubiquitination

**Figure 4:**
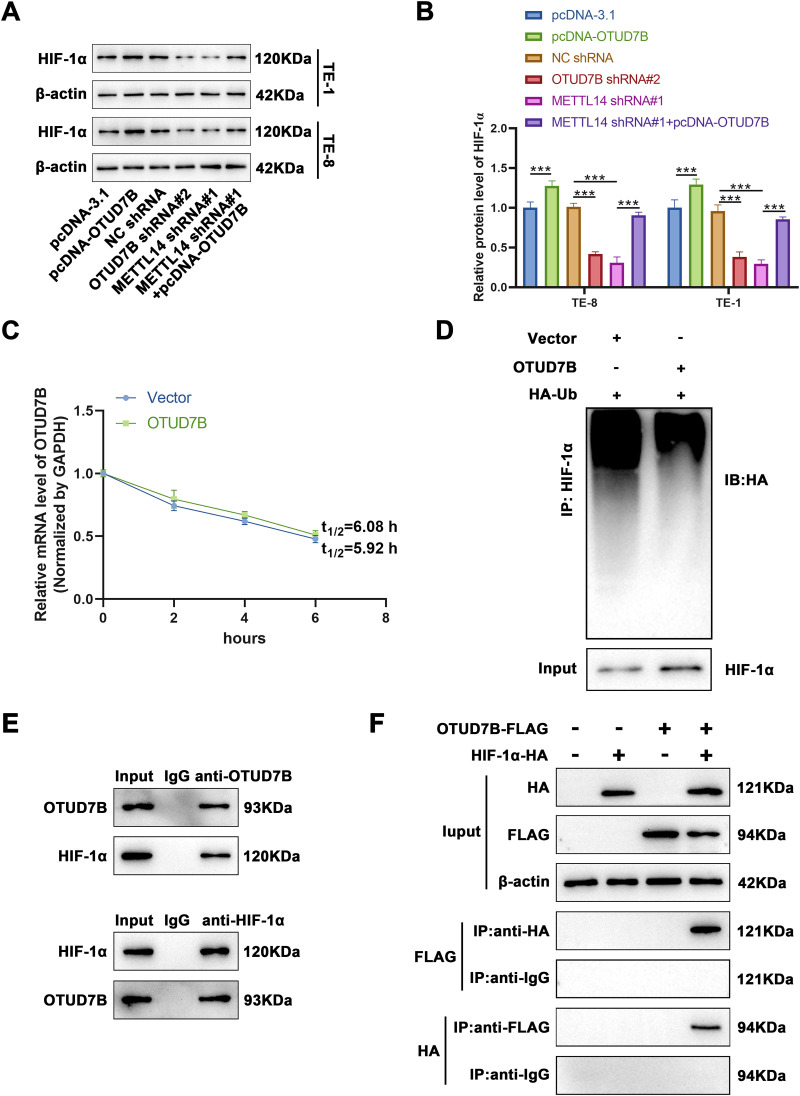
OTUD7B stabilizes HIF-1α expression by modifying deubiquitination. **(A,B)**: Western blotting-based assessment of HIF-1α expression across different cell groups. **(C)**: Determination of the degradation rate of HIF-1α mRNA at various time points by RT-qPCR after overexpression of OTUD7B in TE-8 cells and treatment with Act D (5 μg/mL). **(D)**: Immunoprecipitation evaluation of the ubiquitination status of HIF-1α in TE-8 cells co-transfected with OTUD7B and HA-conjugated ubiquitin constructs. **(E)**: Co-immunoprecipitation assay-based investigation of the interaction between OTUD7B and HIF-1α. **(F)**: Co-immunoprecipitation assay-based confirmation of the direct interaction between OTUD7B and HIF-1α in TE-8 cells transfected with FLAG-tagged OTUD7B or/and HA-conjugated HIF-1α constructs. Data are expressed as mean ± SEM, *n* = 3; ***p < 0.001

The foregoing database analyses indicated a positive correlation between the HIF-1α signaling pathway and OTUD7B expression in ESCC. Accordingly, the HIF-1α levels were examined in cells from various groups. As shown in Fig. 4A,B, overexpression of OTUD7B led to increased HIF-1α expression, while OTUD7B knockdown had the opposite effect. Furthermore, METTL14 knockdown downregulated HIF-1α, which was partially reversed by OTUD7B overexpression. Given that OTUD7B is a deubiquitinating enzyme, Act D treatment combined with RT-qPCR was used to determine whether OTUD7B impacts the stability of HIF-1α mRNA. OTUD7B had no effect on the stability of HIF-1α mRNA (Fig. 4C). These results supported the view that HIF-1α may be functionally related to METTL14/OTUD7B. To verify this, tagged antibody was used to explore whether OTUD7B has an impact on the ubiquitination of HIF-1α. OTUD7B and hemagglutinin-conjugated ubiquitin were transfected into TE-8 cells. Investigation of the ubiquitination level of HIF-1α by IP revealed that OTUD7B increased HIF-1α expression by deubiquitination (Fig. 4D). Next, Co-IP assays were performed to evaluate the interactions between the endogenous OTUD7B and HIF-1α. Endogenous HIF-1α co-immunoprecipitated with OTUD7B, while OTUD7B co-immunoprecipitated with HIF-1α. These findings support the interaction between OTUD7B and HIF-1α (Fig. 4E). To confirm this interaction, HA-HIF-1α and FLAG-OTUD7B were ectopically expressed in TE-8 cells and the cell lysate was subjected to IP using anti-FLAG/HA antibody. HA-HIF-1α co-immunoprecipitated with FLAG-OTUD7B, while the reciprocal Co-IP also confirmed the association of HIF-1α with OTUD7B. These findings definitively established the interaction between OTUD7B and HIF-1α (Fig. 4F). Therefore, it can be concluded that OTUD7B can stabilize the expression of HIF-1α by modifying deubiquitination.

## 4 Discussion

ESCC imposes a significant global health burden with a complex pathophysiology requiring further investigation [18]. In recent years, the emerging field of epigenetics has provided new insights into cancer biology. One epigenetic modification, m6A methylation, has been implicated in various aspects of cancer development and progression [19]. Methylation of m6A is the most common internal mRNA modification in eukaryotic organisms. This methylation influences mRNA stability, translation efficiency, and splicing. Alterations in m6A methylation patterns have been associated with the tumorigenesis and metastasis of ESCC. The m6A modification is markedly enriched in ESCC cells (compared to normal cells) [13]. This heightened presence is largely attributed to the elevated expression of m6A methyltransferases METTL3 and METTL14 and their cofactors within ESCC cells, all of which synergistically contribute to the m6A modification of RNA. m6A methylation is catalyzed by a set of enzymes, including METTL14, which has been identified as a key player in modulating m6A methylation levels [20]. METTL14-mediated m6A methylation affects the expression of numerous genes, thereby influencing cancer cell behavior. METTL14, a central player within the m6A methyltransferase complex, acts synergistically with METTL3 and WTAP to catalyze the m6A methylation of RNA molecules. This modification is pivotal in modulating RNA stability and translation, and is integral to key cellular activities, including cell proliferation and invasiveness [21]. Typically, METTL14 expression is low in tumors and is negatively correlated with patient outcomes. METTL14 reportedly curbs the metastasis of colorectal cancer cells by inhibiting the m6A modification of SRY-box transcription factor 4 (SOX4) mRNA, a mechanism reliant on the YTHDF2-mediated mRNA degradation pathway [22]. Furthermore, METTL14 can impede the migration and invasion of colorectal cancer cells by regulating the demethylation of H3K4me3 [23]. In endometrial cancer, METTL14 acts as a tumor suppressor, and its expression is significantly diminished and is positively associated with tumor aggressiveness and metastatic potential [24]. Conversely, in lung adenocarcinoma, METTL14 overexpression augments tumor cell proliferation, migration, and invasion, whereas its knockdown exerts the opposite effect [25]. Additionally, Wang et al. [26] demonstrated that the increase in METTL14 levels through m6A modification results in reduced levels of PERP, which in turn fosters the growth and metastasis of pancreatic cancer. These findings suggest that the role of METTL14 varies across cancer types.

Within the scope of our research, we focused on the function of OTUD7B, a gene that was identified through bioinformatics analysis as being significantly m6A-enriched in ESCC tissues. Our initial exploration of key genes with m6A enrichment in EC tissues using the GSE179267 dataset revealed 5455 differentially m6A-enriched genes, of which 2679 were m6A-enriched. Among these, OTUD7B emerged as the gene of interest. Further analyses using multiple databases, including TCGA and CCLE, confirmed the upregulation of OTUD7B expression in ESCC tissues and cell lines. A SRAMP database search for OTUD7B mRNA sequences uncovered several m6A binding sites, indicating a potential role for m6A methylation in regulating OTUD7B expression. Previous research has demonstrated that OTUD7B enhances the growth of breast cancer cells by stabilizing estrogen receptor α (ERα) and increasing its expression. Moreover, studies have shown that depletion of OTUD7B markedly reduces the proliferation and migration capabilities of ERα-positive breast cancer cells, whereas overexpression of ERα can counteract the inhibitory effects induced by OTUD7B depletion [27]. Wang et al. [15] revealed that the depletion of OTUD7B markedly impairs the proliferative and sphere-forming capabilities of MDA-MB-468, MDA-MB-453, and MCF7 breast cancer cells, which is attributed to the decreased polyubiquitination of FOXM1 within these cells. OTUD7B also plays a role in the modulation of breast cancer proliferation signals by enhancing the expression of lysine-specific demethylase 1, thereby laying the theoretical groundwork and offering experimental support for the development of treatments and pharmaceuticals aimed at combating breast tumorigenesis [28]. OTUD7B facilitates lung cancer cell metastasis via the Akt/vascular endothelial growth factor signaling pathway [29]. Eliminating OTUD7B significantly reduced the incidence of spontaneous lung cancer in mice, and elevated OTUD7B expression in lung adenocarcinoma tissues has been positively correlated with poor lung cancer prognosis [30]. Collectively, these findings indicate that OTUD7B is upregulated in cancer and contributes to accelerated tumor growth, aligning with poor prognostic outcomes, substantiating the results of this study.

The relationship between OTUD7B and m6A methylation was investigated by examining its association with METTL14. A significant positive correlation was evident between OTUD7B and METTL14 in EC tissues, suggesting that METTL14 may modulate the expression of OTUD7B via the m6A methylation pathway. Indeed, our experiments revealed that METTL14 enhances the mRNA stability and protein expression of OTUD7B by regulating m6A methylation. Knockdown of METTL14 using shRNAs significantly reduced both the protein and mRNA levels of OTUD7B and decreased its m6A modification levels. METTL14 knockdown shortened the half-life of OTUD7B mRNA, further supporting the role of METTL14 in maintaining OTUD7B expression. These results reveal that METTL14 can stabilize the expression of OTUD7B by mediating m6A methylation. Given the observed relationship between OTUD7B and METTL14, we explored the functional consequences of this interaction on EC cell behavior. Our findings revealed that the overexpression of OTUD7B mitigated the inhibitory effects of METTL14 knockdown on cell growth and invasiveness. This suggests that OTUD7B plays an important role in maintaining the aggressive phenotype of EC cells and that its expression is regulated by METTL14-mediated m6A methylation.

Hypoxia within the tumor microenvironment is a hallmark of many solid tumors, including ESCC [31]. The hypoxic environment induces the stabilization and activation of HIF-1α, a key transcription factor that regulates the cellular response to low oxygen conditions. It is widely accepted that HIF-1α governs the activities of a broad range of genes involved in critical processes that facilitate angiogenesis, metabolic reprogramming, invasion, metastasis, and therapy resistance in ESCC [32,33]. A recent study reported that HIF-1α can upregulate programmed death-ligand 1, a protein that helps tumors evade immune detection by suppressing T-cell activity, thereby promoting immune evasion and contributing to tumor progression in ESCC [34]. Herein, database analysis predicted a positive correlation between OTUD7B and HIF-1α, which was further confirmed by experimental results. Overexpression of OTUD7B led to increased expression of HIF-1α, while OTUD7B knockdown had the opposite effect. Moreover, we demonstrated that OTUD7B stabilizes HIF-1α protein expression by modifying deubiquitination. This discovery implies that OTUD7B potentially participates in modulating the HIF-1α signaling pathway, a key regulator in the survival, proliferation, and metastatic spread of cancer cells under hypoxic conditions. HIF-1α exhibits widespread overexpression in EC tissues and positively correlates with tumor invasion depth, distant metastasis, and microvessel density. These significant associations imply that the expression of HIF-1α is likely intimately linked to the invasiveness and prognostic outcomes of EC [35]. In ESCC, HIF-1α expression is implicated in tumor growth, invasion, and metastasis [36]. Additionally, it is closely associated with lymph node metastasis and histological grade [37]. Notably, positive HIF-1α expression is one of the indicators of poor prognosis in ESCC patients, with high expressers exhibiting reduced sensitivity to radiotherapy [38]. These findings position HIF-1α as a promising prognostic biomarker in esophageal carcinoma (EC). Current evidence reveals a critical oxygen-dependent regulatory mechanism: under normoxic conditions, prolyl hydroxylase-mediated hydroxylation of proline residues 402 and 564 in HIF-1α facilitates its recognition by the VHL-containing E3 ubiquitin ligase complex, marking it for proteasomal degradation [39]. This oxygen-sensitive post-translational modification maintains basal HIF-1α levels in well-oxygenated tissues. Conversely, hypoxic conditions disrupt this degradation pathway by inhibiting hydroxylation, leading to HIF-1α stabilization and subsequent activation of its transcriptional program, which drives adaptive responses to oxygen deprivation [40]. Under hypoxic conditions, HIF-1α orchestrates the transcription of several genes involved in angiogenesis, tumor growth, invasion, and metastasis. Upregulation of these genes fuels the progression and aggravation of EC [34]. The relationship between OTUD7B and HIF-1α primarily hinges on the OTUD7B-mediated regulation of HIF-1α stability and function. OTUD7B can remove ubiquitin chains from HIF-1α, preventing its proteasomal degradation and thus sustaining HIF-1α levels and activity. This regulatory mechanism is proteasome-independent, as OTUD7B can effectively modulate HIF-1α stability even in the absence of proteasome activity [41]. Moreover, although HIF-1α degradation depends on the tumor suppressor pVHL, OTUD7B-mediated regulation of HIF-1α does not rely on hydrolase or proteasome activity [41]. These observations suggest that OTUD7B possesses a unique mechanism in governing HIF-1α, potentially involving alternative pathways or molecular mechanisms.

**Figure 5:**
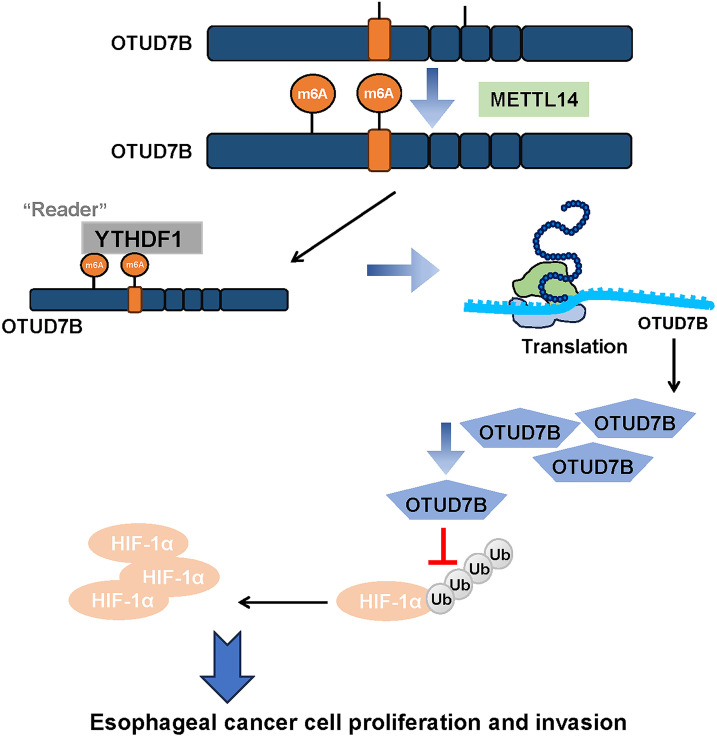
A schematic model summarizing the proposed METTL14-OTUD7B-HIF-1α axis and its role in ESCC progression

This study focused on ESCC, exploring the role of OTUD7B and its relationship with m6A methylation and the HIF-1α signaling pathway. Bioinformatics and experimental data identified OTUD7B as a key m6A-enriched gene in ESCC and the positive correlation of the gene with METTL14 and HIF-1α. METTL14 regulates OTUD7B via m6A methylation, which affects its expression and stability. OTUD7B overexpression counteracts the effects of METTL14 knockdown on cell proliferation and invasion. OTUD7B also stabilizes HIF-1α through deubiquitination, suggesting its role in the HIF-1α pathway. These collective findings demonstrate that METTL14 is capable of facilitating the m6A methylation-mediated stabilization of OTUD7B, which in turn suppresses the ubiquitin-proteasomal degradation of HIF-1α in ESCC (Fig. 5). Clinically, the ability to modulate this axis could lead to the development of novel biomarkers for early ESCC diagnosis. As METTL14 and OTUD7B influence the aggressive phenotypes of ESCC cells, their expression profiles could be used to predict which patients are at higher risk of developing advanced ESCC, allowing for more personalized preventive interventions. Future clinical studies should focus on assessing the efficacy of drugs targeting this axis to improve ESCC prognosis.

This study had some limitations. First, research was predominantly conducted at the cellular level. Although cellular experiments offer invaluable information regarding the functions of genes and proteins, these findings cannot be confirmed *in vivo* in animal models or human subjects. The *in vivo* environment is considerably more complex, potentially encompassing factors not accounted for in cell-based assays, such as the influence of the immune system, complexity of the tumor microenvironment, and pharmacokinetics of drugs. Consequently, whether discoveries made at the cellular level can be directly applied in clinical practice remains uncertain. Second, while the study focused on the interplay between OTUD7B, METTL14, and HIF-1α, it is important to recognize that cancer progression is a multifaceted process involving intricate interactions among multiple genes and factors. There may be unidentified genes or signaling pathways that interact with OTUD7B, thereby influencing its role in ESCC. Furthermore, although we have preliminary data that the m6A site at 2762 in OTUD7B may interact with YTHDF1 to regulate OTUD7B mRNA stability and translation efficiency, our understanding of the overarching regulatory network of m6A methylation in ESCC may be incomplete, highlighting the need for further investigation of other m6A-related enzymes and their possible connections with OTUD7B. Lastly, despite the identification of OTUD7B’s role in stabilizing HIF-1α protein expression through deubiquitination, the precise molecular mechanisms and key regulatory sites involved in this process are not fully understood. How OTUD7B discerns and binds to HIF-1α for deubiquitination, and whether other cofactors are involved, warrant additional research.

While this study provides a solid foundation, several important next steps should be considered to further advance our understanding of the METTL14-OTUD7B-HIF-1α axis and its role in ESCC. Future studies should explore the specific molecular interactions between METTL14, OTUD7B, and HIF-1α *in vivo*, to validate these findings in a more physiologically relevant context. Additionally, clinical studies are needed to assess the expression levels of METTL14, OTUD7B, and HIF-1α in patient populations at risk for advanced ESCC, which could lead to the identification of biomarkers for early detection and risk stratification. Lastly, while the METTL14-OTUD7B-HIF-1α axis represents a promising therapeutic target for ESCC, it is important to consider potential challenges associated with targeting this pathway therapeutically. However, a key concern in molecular pathway targeting is the risk of off-target effects. Future research should not only focus on the efficacy of targeting this pathway but also on ensuring safety profiles that minimize unintended consequences.

## 5 Conclusion

Altogether, our present study demonstrates that METTL14 is capable of facilitating the m6A methylation-mediated stabilization of OTUD7B, which in turn suppresses the ubiquitin-proteasomal degradation of HIF-1α in ESCC.

## Supplementary Materials

Figure S1 Data-based Prediction Discovery of m6A Binding Sites on OTUD7B mRNA Sequence details of the m6A binding sites located at site1829, site1856, site 2146, and site 2762 are provided.

Figure S2 Identification of m6A Modification Sites and Determination of their Roles in OTUD7B Stability and Translation Efficiency. **A**: Gene-specific m6A-PCR evaluation of the m6A modification levels on the OTUD7B transcript in TE-8 cells transfected with WT, 1829mut, 1856mut, 2146mut, and 2762mut. **B-C**: OTUD7B expression assessed by qRT-PCR (**B**) and western blotting (**C**) in cells transfected with WT, 1829mut, 1856mut, 2146mut, and 2762mut. **D**: Determination of the degradation rate of OTUD7B mRNA at various time points by RT-qPCR after mutation of site 2762 in OTUD7B of TE-8 cells, followed by treatment with actinomycin D (Act D, 5 μg/mL). **E**: Co-immunoprecipitation-based confirmation of the direct interaction between OTUD7B and YTHDF1 in TE-8 cells transfected with FLAG-tagged OTUD7B or FLAG-tagged OTUD7B 2762mut along with HA-tagged YTHDF1. Data are expressed as mean ± SEM, n=3; **p＜0.01,***p＜0.001.

## Data Availability

All data generated or used during the study appear in the submitted article.

## Supplementary Materials

The supplementary material is available online at https://www.techscience.com/doi/10.32604/or.2025.061301/s1.

## Abbreviations

OTUD7B: Ovarian tumor deubiquitinase 7B
EC: Esophageal cancer
ESCC: Esophageal squamous cell carcinoma
EAC: Esophageal adenocarcinoma
HIF-1α: Hypoxia-inducible factor-1α
m6A: N6-methyladenosine
m1A: N1-methyladenosine
m7G: N7-methylguanosine
m5C: 5-Methylcytosine
GEO: Gene Expression Omnibus
FBS: Fetal bovine serum
ATCC: American Type Culture Collection
DEG: Differentially expressed genes
Co-IP: Co-immunoprecipitation
SEM: Standard error of the mean
ANOVA: Analysis of variance
YTHDF2: YTH N6-methyladenosine RNA binding protein 2
