# Supplementary figures and images for: OTUD7B Stabilization by METTL14-Mediated m6A Methylation Drives HIF-1α Expression in Esophageal Squamous Cell Carcinoma
Source: Oncol Res. 2025 Jul 18;33(8):2055–74. doi: 10.32604/or.2025.061301 (PMC12308248; doi:10.32604/or.2025.061301)

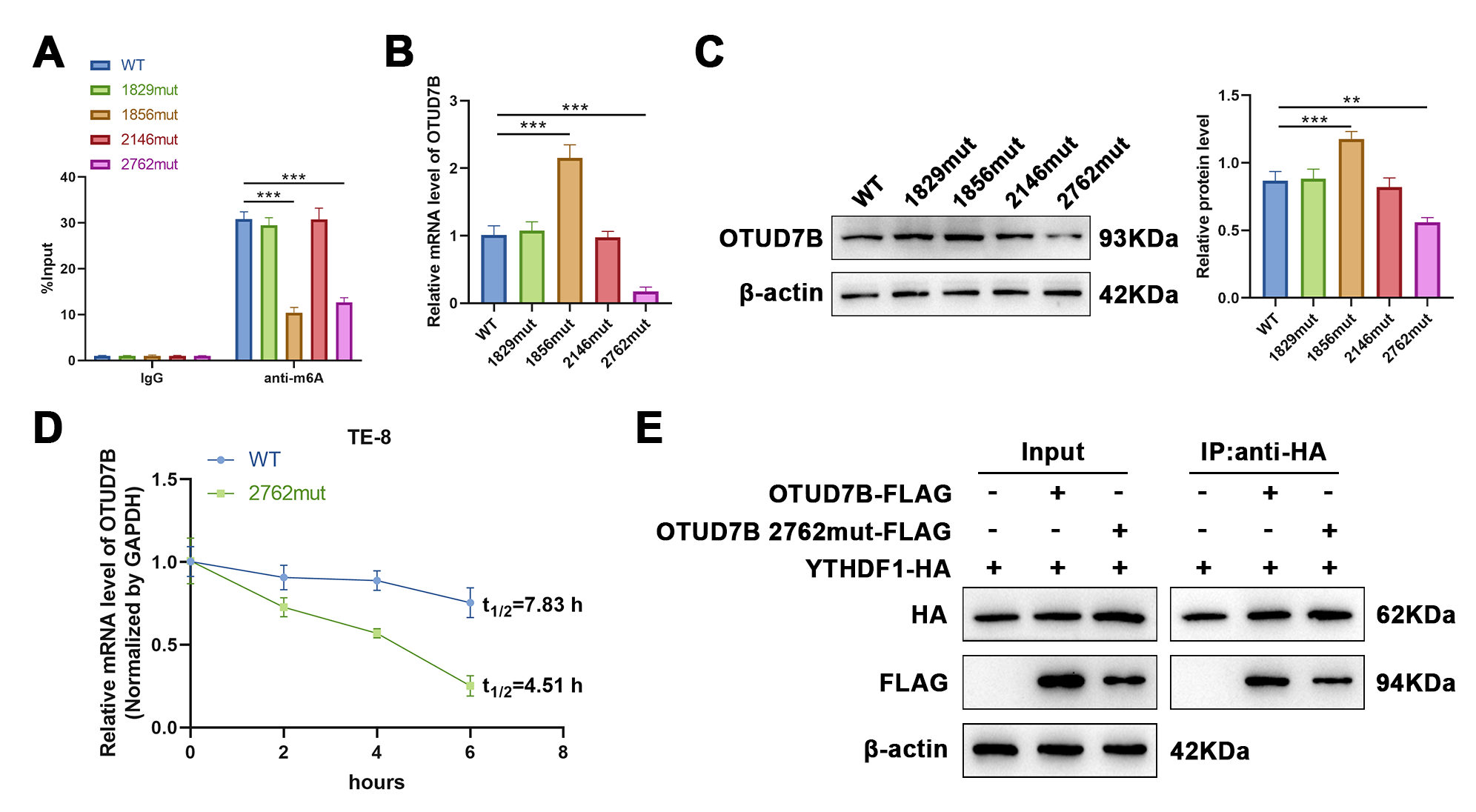

Supplement: Figure S2 — A: Gene-specific m6A-PCR evaluation of the m6A modification levels on the OTUD7B transcript in TE-8 cells transfected with WT, 1829mut, 1856mut, 2146mut, and 2762mut. B-C: OTUD7B expression assessed by qRT-PCR (B) and western blotting (C) in cells transfected with WT, 1829mut, 1856mut, 2146mut, and 2762mut. D: Determination of the degradation rate of OTUD7B mRNA at various time points by RT-qPCR after mutation of site 2762 in OTUD7B of TE-8 cells, followed by treatment with actinomycin D (Act D, 5 μg/mL). E: Co-immunoprecipitation-based confirmation of the direct interaction between OTUD7B and YTHDF1 in TE-8 cells transfected with FLAG-tagged OTUD7B or FLAG-tagged OTUD7B 2762mut along with HA-tagged YTHDF1. Data are expressed as mean ± SEM, n=3; **p＜0.01,***p＜0.001. [file OncolRes-33-61301-s002.tif]
